# Road Agglomerate Fog Detection Method Based on the Fusion of SURF and Optical Flow Characteristics from UAV Perspective

**DOI:** 10.3390/e27111156

**Published:** 2025-11-14

**Authors:** Fuyang Guo, Haiqing Liu, Mengmeng Zhang, Mengyuan Jing, Xiaolong Gong

**Affiliations:** School of Transportation and Logistic Engineering, Shandong Jiaotong University, Jinan 250357, China; guofuyang0916@gmail.com (F.G.); zhangmengmeng@sdjtu.edu.cn (M.Z.); mengyuanjing45@gmail.com (M.J.); xiaolonggong17@gmail.com (X.G.)

**Keywords:** UAV-based road monitoring, agglomerate fog detection, SURF, optical flow analysis, Bayesian fusion

## Abstract

Road agglomerate fog seriously threatens driving safety, making real-time fog state detection crucial for implementing reliable traffic control measures. With advantages in aerial perspective and a broad field of view, UAVs have emerged as a novel solution for road agglomerate fog monitoring. This paper proposes an agglomerate fog detection method based on the fusion of SURF and optical flow characteristics. To synthesize an adequate agglomerate fog sample set, a novel network named FogGAN is presented by injecting physical cues into the generator using a limited number of field-collected fog images. Taking the region of interest (ROI) for agglomerate fog detection in the UAV image as the basic unit, SURF is employed to describe static texture features, while optical flow is employed to capture frame-to-frame motion characteristics, and a multi-feature fusion approach based on Bayesian theory is subsequently introduced. Experimental results demonstrate the effectiveness of FogGAN for its capability to generate a more realistic dataset of agglomerate fog sample images. Furthermore, the proposed SURF and optical flow fusion method performs higher precision, recall, and F1-score for UAV perspective images compared with XGBoost-based and survey-informed fusion methods.

## 1. Introduction

Agglomerate fog is a special fog that is much denser and has lower visibility, appearing in the range of tens to hundreds of meters [1]. Since agglomerate fog can severely impair a driver’s vision, it is difficult to promptly discern the road conditions ahead, and it is highly prone to triggering chain-reaction traffic accidents, inducing a significant threat to traffic safety. Traditional agglomerate fog monitoring methods can be classified into two types: (1) active remote sensing technology, including meteorological sensors, LiDAR, infrared imaging devices [2,3], and meteorological satellites [4]; and (2) vision-based detection technology [5,6,7,8,9,10,11,12,13,14]. However, active remote sensing technology faces challenges in signal attenuation, low spatial resolution, coverage constraints, and high implementation costs, which prevent it from being widely adopted in the field of traffic management. Vision-based detection technology can obtain richer feature information for more accurate environment monitoring using existing road surveillance equipment without additional cost investment. Hence, the vision-based methods hold broader application prospects in real-time monitoring of agglomerate fog.

Currently, vision-based research on agglomerate fog mainly focuses on (1) the fog generation and removal methods [5,6], (2) recognition and tracking methods of traffic objects under agglomerate foggy conditions [7,8,9], and (3) the detection of agglomerate fog in the road environment. For the detection of agglomerate fog, the approaches can be classified into traditional image processing methods and AI (artificial intelligence)-based methods. Traditional image processing methods primarily employ image feature extraction and classification approaches to accomplish fog state recognition, such as utilizing gray-level co-occurrence matrix [10,11], edge detection [12], or texture analysis [13,14]. Meanwhile, AI-based methods leverage large-scale patchy fog datasets for model training and perform state classification guided by gradient convergence principles, with predominant models including support vector machines (SVM), clustering analysis, K-nearest neighbor (KNN), and convolutional neural networks (CNN), and other models.

In [15], the authors propose a fog-density-level distinction method using an SVM classification framework that incorporates both global and local fog-related features, such as entropy, contrast, and dark channel information. The method achieves a classification accuracy of 85.8%, with greater robustness compared to traditional image processing methods, such as edge detection or texture analysis. To comparatively analyze the performance of SVM, K-NN, and CNN, the authors in [16] examine the three methods for identifying foggy and clear weather images based on the gray-level co-occurrence matrix extracted from ground-moving onboard scenario images. In [17], the authors propose an Attention-based BiLSTM-CNN (ABCNet) model, which integrates attention mechanisms with BiLSTM and CNN architectures to predict atmospheric visibility caused by heavy fog. Current research shows that artificial intelligence methods, which extract effective image features and convolutional characteristics and construct gradient descent convergence classifiers, can achieve efficient fog classification and atmospheric visibility prediction. This approach has now been a mainstream research direction.

In recent years, unmanned aerial vehicles (UAVs) have been widely applied in traffic detection, including traffic flow status, traffic events, and road environmental conditions, using technologies such as visual analysis, multi-sensor fusion, and edge computing [18,19,20]. In the field of fog research, the authors in [21] employed a classification module and a style migration module to address the challenge of non-uniform dense fog removal in UAV imagery without relying on paired foggy and corresponding fog-free datasets. The proposed method significantly enhanced the visual clarity and interpretability of complex foggy scenes using the classification-guided thick-fog-removal network and style migration mechanism. In [22], a multi-task framework named ODFC-YOLO was proposed, which seamlessly integrated image dehazing into a YOLO detection subnetwork via end-to-end joint training. This combination significantly improved detection accuracy and maintained high real-time performance for UAV-based object recognition in dense fog environments. However, existing methods primarily analyze static scenario images without consideration of the challenges caused by moving UAVs in dynamic flight conditions, such as motion-induced blurring, viewpoint variation, and fog density gradation. Furthermore, in real-world scenarios, the scarcity of agglomerate fog datasets limits model training performance and generalization capability.

To address these challenges, this paper develops a UAV-based framework for agglomerate fog detection, including a FogGAN model for generating agglomerate fog images to alleviate data scarcity, and an agglomerate fog detection model based on Bayesian fusion of SURF and optical flow characteristics for the fog state judgement. In the new UAV aerial perspective, the proposed fusion method integrates both the static and dynamic features of UAV images and performs higher precision, recall, and F1-score compared with traditional approaches.

## 2. Agglomerate Fog Sample Dataset Generation Method Based on GAN

Since agglomerate fog has characteristics of rapid formation and dissipation, limited spatial coverage, and shifting locations with air currents dynamically, it is difficult to acquire substantial valid samples in actual road environments. This paper adopts the generative adversarial network (GAN) [23] to synthesize an agglomerate fog sample dataset based on a limited number of field-collected images.

**Figure 1:**
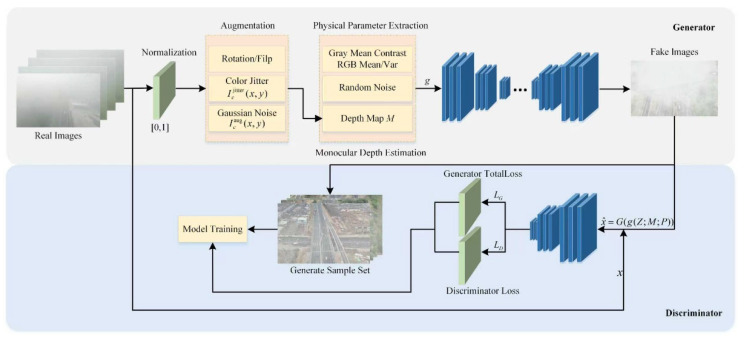
The generation and working principle of GAN model.

To obtain a high-quality and realistic agglomerate fog dataset, this paper proposes an improved generative adversarial network named FogGAN, in which the generator is enhanced by injecting physical image cues into its feature modulation layers for producing agglomerate fog with more authentic tonal and structural characteristics. The architecture of the FogGAN is shown in Figure 1.

### 2.1. Generator

The agglomerate fog image samples acquired from actual road scenarios are linearly normalized to the unit interval [0,1], as expressed by Equation (1).

(1) $${\tilde{I}}_{c}(x,y)=\frac{I_{c}(x,y)-I_{\min}}{I_{\max}-I_{\min}},\;c\in \{R,G,B\}$$

In Equation (1), $I_{\min}$ and $I_{\max}$ are denoted as the minimum and maximum light intensity values of the agglomerate fog image samples, respectively.

To simulate variations in viewpoint and sensor noise characteristics in actual road scenarios, random rotation and horizontal/vertical flipping, color jittering, and Gaussian noise injection are carried out for the augmentation of agglomerate fog images.

The color jittering is expressed by Equation (2).

(2) $$I_{c}^{\mathrm{jitter}}(x,y)={\tilde{I}}_{c}^{\mathrm{jitter}}(x,y)\left(\begin{array}{c} 1+\delta_{c} \end{array}\right)\;\;\;\;\;\;\;\;\delta_{c}\sim U(-0.1,0.1)$$

In Equation (2), $I_{c}^{\mathrm{jitter}}(x,y)$ is the color jittering image. $\delta_{c}$ represents independent perturbations. Color jittering modifies image properties, including brightness, contrast, and saturation.

The Gaussian noise is expressed by Equation (3):

(3) $$I_{c}^{\mathrm{aug}}(x,y)=I_{c}^{\mathrm{jitter}}(x,y)+\eta_{c}(x,y)\;\;\;\;\;\;\;\;\eta_{c}\sim N(0,\sigma^{2})$$

In Equation (3), $I_{c}^{\mathrm{aug}}(x,y)$ is the image after adding Gaussian noise. $\eta_{c}(x,y)$ is the random noise following the Gaussian distribution of standard deviation σ, which is generally valued in the range [0.01,0.05].

These augmentations enrich the training set with diverse lighting, color balance, and noise conditions. Based on the augmented image, the depth map describes the distance from each pixel to the camera and is produced by a CNN-based monocular depth estimation model that is pretrained on UAV perspective images.

The global photometric statistics from $\tilde{I}$ are extracted and further converted into a grayscale image following the ITU-R BT.601 [24] standard by Equation (4). In this study, the photometric statistics are computed from the real images to maintain the photometric consistency of the generated images. The grayscale conversion is performed as follows:

(4) $$I_{\mathrm{gray}}(x,y)=0.299{\tilde{I}}_{R}(x,y)+0.587{\tilde{I}}_{G}(x,y)+0.114{\tilde{I}}_{B}(x,y)$$

Referring to Equation (4), the mean gray level is calculated by Equation (5).

(5) $$I_{\mathrm{gray}}^{ave}=\frac{1}{HW}\sum_{x=1}^{H}\sum_{y=1}^{W}I_{\mathrm{gray}}(x,y)$$

where *H* is the height of the image and *W* is the width. Further, the gray-level contrast, i.e., the standard deviation, is calculated by Equation (6).

(6) $$K=\sqrt{\frac{1}{HW}\sum_{x,y}{\left(I_{\mathrm{gray}}(x,y)-I_{\mathrm{gray}}^{ave}\right)}^{2}}$$

For each color channel in c∈{R,G,B}, the average and the standard deviation of light intensity are calculated by Equations (7) and (8), respectively.

(7) $$\mu_{c}=\frac{1}{HW}\sum_{x,y}{\tilde{I}}_{c}(x,y)$$

(8) $$\sigma_{c}=\sqrt{\frac{1}{HW}\sum_{x,y}{\left({\tilde{I}}_{c}(x,y)-\mu_{c}\right)}^{2}}$$

After normalization, the physical parameter vector of the agglomerate fog image sample (8) can be expressed by Equation (9).

(9) $$P={\left[\begin{array}{c} \hat{g},\hat{K},{\hat{\mu }}_{R},{\hat{\mu }}_{G},{\hat{\mu }}_{B},{\hat{\sigma }}_{R},{\hat{\sigma }}_{G},{\hat{\sigma }}_{B} \end{array}\right]}^{T}$$

The depth map *M*, the physical parameter vector *P,* and the random noise vector *Z* collectively form the input of the Generator. Since the proposed method incorporates the physical features of the image samples, FogGAN can ensure that the generated agglomerate fog images exhibit enhanced realism.

In practice, g(Z;M;P) is instantiated as a 1-D latent vector concatenating the embeddings of the depth map *M*, the photometric vector *P* and the noise *Z*. This vectorized input *g* is fed to the fully connected layer in Equation (10).

(10) $$f^{\left(1\right)}=W^{\left(1\right)}g+b^{\left(1\right)}$$

where $W^{\left(1\right)}$ and $b^{\left(1\right)}$ are the weight matrix and bias vector, respectively.

To achieve physics-driven fog attenuation, these features are further modulated using a depth-dependent transmission coefficient $\alpha (M;\theta_{M})$ and a learnable mapping $\beta (P;\theta_{P})$ of the photometric parameters using Equation (11).

(11) $$f^{\left(2\right)}=\alpha \left(\begin{array}{c} M;\theta_{M} \end{array}\right)⊙f^{\left(1\right)}+\beta \left(\begin{array}{c} P;\theta_{P} \end{array}\right)$$

In Equation (11), $\alpha (M;\theta_{M})$ is a per-pixel mask of size 1 × *H* × *W* that multiplies all channels at location (x,y) (element-wise multiplication). $\alpha (M;\theta_{M})$ is calculated using Equation (12).

(12) $$\alpha (M;\theta_{M})=\exp\left(-\kappa \hat{M}\right),\;\hat{M}=\mathrm{normalize}(M)$$

where $\theta_{M}=\kappa$ and κ > 0 control the attenuation strength.

$\beta (P;\theta_{P})$ is a per-channel bias of size *C* × 1 × 1 that is broadcast over H × W. $\beta (P;\theta_{P})$ is calculated using Equation (13).

(13) $$\beta (P;\theta_{P})=\mathrm{MLP}(P)\in R^{C\times 1\times 1}$$

where $\theta_{P}$ denotes the learnable parameters of the MLP (weights and biases).

**Figure 2:**
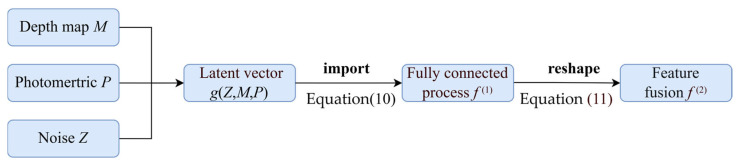
Physical cue injection process.

As mentioned in the aforementioned analysis, the physical cues injection process is shown in Figure 2.

The modulated feature $f^{\left(2\right)}$ is passed through *L* successive up-sampling blocks, which consist of convolution, BatchNorm, and ReLU activation, as shown in Equation (14).

(14) $$f^{\left(l+1\right)}=\mathrm{ReLU}\left(\mathrm{BatchNorm}(\mathrm{Conv}(f^{\left(l\right)}))\right),\;l=2,\ldots ,L-1$$

The output fake images are expressed by Equation (15).

(15) $$\hat{x}=\mathrm{TConv}(f^{\left(L\right)})$$

The entire generator is trained to minimize the combined loss as given by Equation (16).

(16) $$\left\{\begin{array}{l} L_{G}=L_{\mathrm{adv}}+\lambda_{\mathrm{phy}}L_{\mathrm{phy}}+\lambda_{\sup}L_{\sup}\\ L_{\mathrm{adv}}=E_{Z,M,P}[-\log D(\hat{x})]\\ L_{\mathrm{phy}}={\left\|B(\hat{x})-P\right\|}_{2}^{2}\\ L_{\sup}={\left\|\hat{x}-x\right\|}_{1} \end{array}\right.$$

where D(⋅) is the discriminator, and B(⋅) extracts the same global photometric statistics defined in Equations (4)–(9). $E_{Z,M,P}$ denotes expectation over Z~p(z), and $(M,P)\sim p_{\mathrm{data}}$, $\lambda_{\mathrm{phy}}$, and $\lambda_{\sup}$ are the weights.

### 2.2. Discriminator

Taking either the real foggy image x or the fake foggy image $\hat{x}=G(g(Z;M;P))$ created by the Generator as input, a scalar D(⋅)∈[0,1] is produced to describe the reality probability. The discriminator architecture consists of *Q* down-sampling blocks and a fully connected layer, as expressed by Equations (17)–(19).

(17) $$h^{\left(0\right)}=x\;\mathrm{or}\;\hat{x}$$

(18) $$h^{\left(q+1\right)}=\mathrm{LeakyReLU}\left(\mathrm{BatchNorm}({\mathrm{Conv}}^{\left(q\right)}(h^{\left(q\right)}))\right)\;q=0,\ldots ,Q-1$$

(19) $$D(x)=S\left(W^{\left(e\right)}h^{\left(Q\right)}+b^{\left(e\right)}\right)$$

where each ${\mathrm{Conv}}^{\left(q\right)}$ is a convolution with stride 2. $W^{\left(e\right)}$ and $b^{\left(e\right)}$ are the weights and bias of the final linear layer. S is the sigmoid activation.

The discriminator is trained to minimize the standard binary-cross-entropy loss as given by Equation (20).

(20) $$L_{D}=-E_{x\sim p_{\mathrm{data}}}\left[\log D(x)\right]-E_{g\sim p_{g}}\left[\log\left(1-D(G(g(Z;M;P)))\right)\right]$$

By alternately minimizing $L_{D}$ with respect to D and minimizing the generator loss $L_{G}$ with respect to G, the two networks play the minimax game that drives G to produce increasingly realistic foggy images.

## 3. Dense Fog Detection Method Based on SURF Feature Extraction and Optical Flow Analysis

### 3.1. ROI Division for UAV Perspective

The agglomerate fog has characteristics of small spatial scale and localized distribution, whereas the forward-facing perspective of drones during UAV cruise has a broad distant view. It is imperative to define the region of interest (ROI) for fog detection, which is beneficial for accurately locating where the fog occurs and for reducing computational overhead. In this paper, trapezoidal ROI along the road within different image depth ranges are preset and applied for further research.

**Figure 3:**
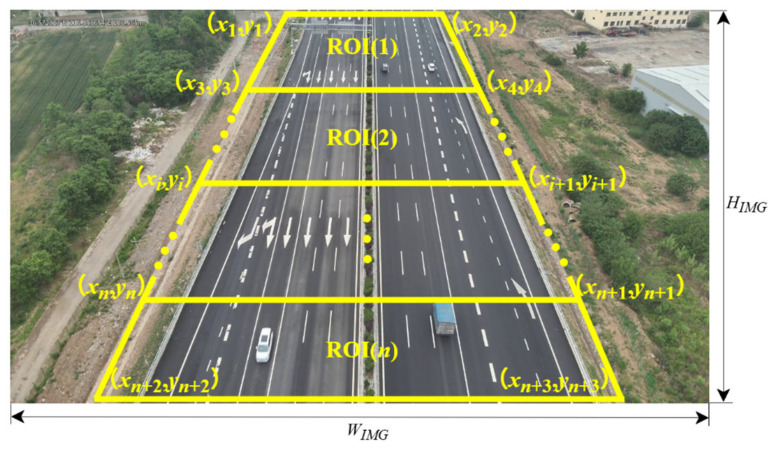
Distribution of regions of interest.

For the image resolution of $W_{IMG}\times H_{IMG}$, *n* trapezoidal ROIs are divided according to Equation (21).

(21) $$ROI(i)=\left\{\begin{array}{l} \left(x_{i,1},y_{i,1})=(\frac{\left(n-i\right)x_{\mathrm{TL}}+\left(i-1\right)x_{\mathrm{BL}}}{n-1},\frac{i-1}{n}H_{IMG}\right)\\ \left(x_{i,2},y_{i,2})=(\frac{\left(n-i\right)x_{\mathrm{TR}}+\left(i-1\right)x_{\mathrm{BR}}}{n-1},\frac{i-1}{n}H_{IMG}\right)\\ \left(x_{i,3},y_{i,3})=(\frac{\left(n-i\right)x_{\mathrm{TR}}+\left(i-1\right)x_{\mathrm{BR}}}{n-1},\frac{i}{n}H_{IMG}\right)\\ (x_{i,4},y_{i,4})=(\frac{\left(n-i\right)x_{TL}+\left(i-1\right)x_{BL}}{n-1},\frac{i}{n}H_{IMG})\;i,j=1,2,\ldots ,n \end{array}\right.$$

where $\left(x_{i,j},y_{i,j}\right)$ are the pixel coordinates of the *j*-th vertex of the *i*-th ROI (*j* = 1, 2, …, *n*; *i* = 1, 2, …, *n*); $x_{\mathrm{TL}}$, $x_{\mathrm{BL}}$, $x_{\mathrm{TR}}$, and $x_{\mathrm{BR}}$ denote the horizontal pixel positions of the trapezoid’s top-left, top-right, bottom-left, and bottom-right base points, respectively; and $W_{IMG}$ and $H_{IMG}$ are the image width and height as shown in Figure 3.

### 3.2. SURF Extraction of Road Agglomerate Fog

For each ROI, the SURF (speeded-up robust features) [25] algorithm is employed for robust detection and description of interest points. The integral image at any location (*x*,*y*) is defined as the cumulative sum of pixel intensities above and to the left of (*x*,*y*), denoted by Equation (22).

(22) $$I_{\Sigma }(x,y)=\sum_{i=0}^{x}\sum_{j=0}^{y}I(i,j)$$

Based on the integral image acquired by Equation (22), box filters are applied to approximate the second-order Gaussian derivatives $L_{xx}(x,y,s)$, $L_{xy}(x,y,s)$, and $L_{yy}(x,y,s)$ at scale *s*. The resulting responses are combined into the Hessian matrix, as expressed by Equation (23).

(23) $$\mathrm{Hessian}(x,y,s)=\left[\begin{array}{cc} L_{xx}(x,y,s) & L_{xy}(x,y,s)\\ L_{xy}(x,y,s) & L_{yy}(x,y,s) \end{array}\right]$$

As shown in Equation (23), the determinant of this Hessian(x,y,s) effectively approximates the response of the Laplacian-of-Gaussian operator. In this paper, the points with Hessian(x,y,s) values above the threshold in both spatial and scale dimensions are defined as key points.

**Figure 4:**
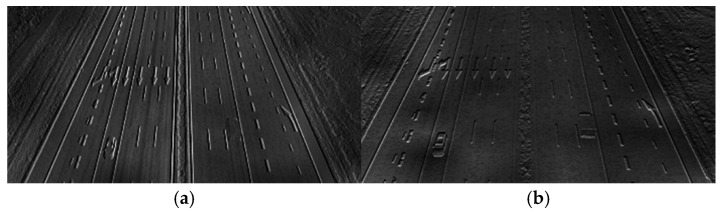
Haar responses of the road scenario: (**a**) Horizontal Haar response *H_x_*; (**b**) Vertical Haar response *H_y_*.

The orientation of each feature point is assigned using the Haar wavelet transform on the circular neighborhood around the key point. Figure 4 shows the differentiated response of the Haar wavelet in the *x* and *y* directions in the same road scenario.

The wavelet responses are aggregated in both horizontal and vertical directions, and the orientation θ is acquired using Equation (24).

(24) $$\theta =\mathrm{atan}2\left(\sum H_{y},\sum H_{x}\right)$$

where $w_{x}$ and $w_{y}$ denote the Haar wavelet transform in the horizontal and vertical directions, respectively, and atan2 is the four-quadrant inverse tangent function.

**Figure 5:**
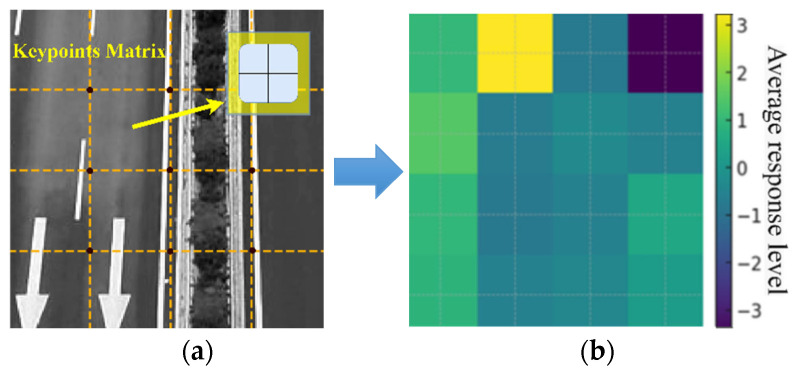
Construction of SURF descriptors: (**a**) 4 × 4 subregion response matrix; (**b**) Mean horizontal response per subregion.

Finally, to construct the SURF descriptor, a square region centered at each key point is divided into a 4 × 4 grid of subregions, as shown in Figure 5a. Within each of the 4 × 4 subregions, four statistical values of the Haar responses are accumulated. Concatenating these four values from all 16 subregions yields a 64-D descriptor, as shown in Figure 5b.

After detection, the count of SURF key points within the region of interest is denoted by $N_{f}$ for the *i*-th ROI and is used as one evidential variable in the subsequent Bayesian fusion.

### 3.3. Optical Flow Analysis of Road Agglomerate Fog

The SURF algorithm is primarily applied for static feature extraction of individual images. To improve the accuracy of agglomerate fog detection during UAV adaptive cruise, this paper further integrates optical flow features for dynamic feature extraction. The Lucas–Kanade [26] algorithm is used to capture dynamic features of agglomerate fog by pixel motion across consecutive image frames for each ROI.

Referring to the brightness constancy assumption, pixel values exhibit minimal variation over short time intervals between consecutive frames, as expressed by Equation (25).

(25) I(x,y,t)=I(x+Δx,y+Δy,t+Δt)

Using the first-order Taylor series, the optical flow constraint equation is obtained and expressed by Equation (26).

(26) $$I_{x}u+I_{y}v+I_{t}=0$$

In Equation (26), $I_{x}$, $I_{y}$, and $I_{t}$ are partial derivatives of image intensity in the spatial (*x*,*y*) and temporal *t* domains. *u* and *v* represent horizontal and vertical components of the optical flow vector, respectively.

By establishing an overdetermined system of equations using multiple neighboring pixels, Equation (26) can be further transformed into Equation (27), and the solution of the optical flow constraint equation can be treated as a least squares problem.

(27) $$\left[\begin{array}{cc} \sum I_{x}^{2} & \sum I_{x}I_{y}\\ \sum I_{x}I_{y} & \sum I_{y}^{2} \end{array}\right]\left[\begin{array}{c} u\\ v \end{array}\right]=-\left[\begin{array}{c} \sum I_{x}I_{t}\\ \sum I_{y}I_{t} \end{array}\right]$$

Referring to the previously identified SURF key points of consecutive frames, optical flow vectors are computed for capturing displacement and directionality of feature movements. In agglomerate fog areas, atmospheric particles typically exhibit subtle and coherent motion, resulting in smaller magnitudes and directionally consistent optical flow vectors. In contrast, non-fog areas tend to display irregular and diverse motion features.

Let $w(x,y,t)={\left[u(x,y,t),v(x,y,t)\right]}^{T}$ denote the Lucas–Kanade flow vector at pixel (*x*,*y*) between two consecutive frames *t* and *t* + 1. Its magnitude is expressed by Equation (28).

(28) $$M_{v}=\|w(x,y,t){\|}_{2}=\sqrt{u{\left(x,y,t\right)}^{2}+v{\left(x,y,t\right)}^{2}}$$

The mean magnitude of optical flow vectors is denoted by $M_{v}$ for the *i*-th ROI and is used as the other evidential variable in the subsequent Bayesian fusion.

### 3.4. Bayesian-Based Data Fusion for Agglomerate Fog Detection

For each ROI in the frame of the road image detected by UAV, calculate the number of SURF $N_{f}$ and the mean optical flow magnitude $M_{v}$. The likelihoods under the “there is agglomerate fog in the ROI” hypothesis are expressed by Equations (29) and (30), respectively.

(29) $$p_{f}\left(N_{f}|\mathrm{ROI}\right)=\frac{1}{\sqrt{2\pi }\sigma_{f}}\exp\left(-\frac{{\left(N_{f}-\mu_{f}\right)}^{2}}{2\sigma_{f}^{2}}\right)$$

(30) $$p_{m}(M_{v}|\mathrm{ROI})=\frac{1}{\sqrt{2\pi }\sigma_{m}}\exp\left(-\frac{{\left(M_{v}-\mu_{m}\right)}^{2}}{2\sigma_{m}^{2}}\right)$$

where $\mu_{f}$ and $\sigma_{f}$ denote the mean and standard deviation of SURF counts, respectively. $\mu_{m}$ and $\sigma_{m}$ are the mean and standard deviation of motion magnitudes, respectively. These parameters are derived from statistical analysis of the samples by FogGAN.

Assuming the prior agglomerate fog probability $P_{0}$ is conditionally independent of $N_{f}$ and $M_{v}$, the posterior probability of agglomerate fog can be expressed by Equation (31).

(31) $$P(\mathrm{fog}|N_{f},M_{v},\mathrm{ROI})=\frac{P_{0}p_{f(N_{f}|\mathrm{ROI})}p_{m}(M_{v}|\mathrm{ROI})}{P_{0}p_{f(N_{f}|\mathrm{ROI})}p_{m}(M_{v}|\mathrm{ROI})+(1-P_{0})\left[\begin{array}{c} 1-p_{f(N_{f}|\mathrm{ROI})} \end{array}\right]\left[1-p_{m}(M_{v}|\mathrm{ROI}\right]}$$

When the posterior probability is larger than the preset threshold τ, it can be considered that agglomerate fog has occurred, as expressed by Equation (32).

(32) $$P\left(\mathrm{fog}|N_{f},M_{v},\mathrm{ROI}\right)\geq \tau$$

## 4. Experimental Results and Analysis

### 4.1. Experimental Conditions

**Figure 6:**
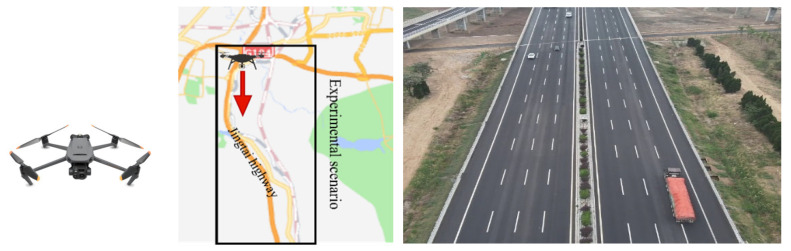
UAV and experimental scenario.

In this paper, the DJI Mavic 3T UAV is used for original agglomerate fog image collection, and the configurations presented in Table 1 are collected from mileage post number K470 to K480 of the Jingtai Highway. The road scenario is shown in Figure 6.

### 4.2. FogGAN-Based Dataset Generation Results and Analysis

**Figure 7:**
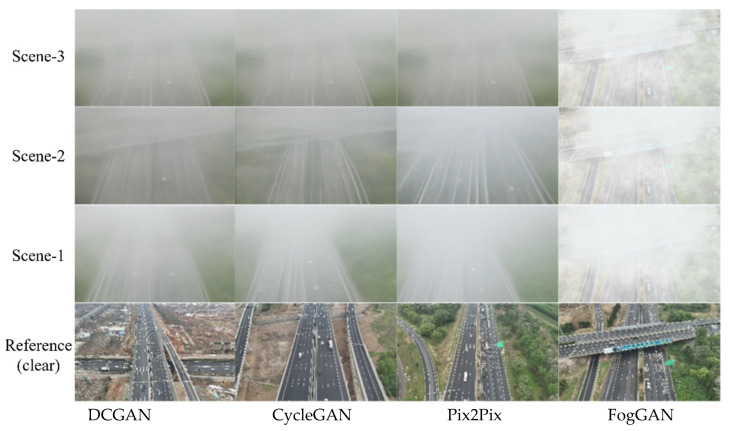
Agglomerate fog visual effects using different methods.

To evaluate the performance of the proposed FogGAN, comparative experiments were conducted against three benchmark methods: DCGAN [27], CycleGAN [28], and Pix2Pix [29]. The visual effects of agglomerate fog images generated by different methods are presented in Figure 7.

To carry out a quantitative evaluation of the results generated by different methods, this paper applies three indexes for further comparative analysis, including peak signal-to-noise ratio (*PSNR*), structural similarity index (*SSIM*), and Frechet inception distance (*FID*). The indexes are calculated as follows:

① *PSNR*: Generated images with a greater *PSNR* have higher fidelity to the reference. The PSNR is calculated by Equation (33).

(33) $$PSNR=10\log_{10}\left(\frac{{\left({\mathrm{MAX}}_{I}\right)}^{2}}{MSE}\right)$$

where MAX_I_ is the maximum possible pixel value. *MSE* is the mean squared of the referenced image *X* and the generated image *Y*, as expressed by Equation (34).

(34) $$MSE=\frac{1}{3H_{IMG}W_{IMG}}\sum_{i=1}^{H_{IMG}}\sum_{j=1}^{W_{MG}}\sum_{k=1}^{3}{\left(X(i,j,k)-Y(i,j,k)\right)}^{2}$$

② *SSIM*: Generated images with a greater *SSIM* mean they are closer to the reference in luminance, contrast, and structure. The *SSIM* is calculated using Equation (35).

(35) $$SSIM(X,Y)=\frac{\left(2\mu_{X}\mu_{Y}+V_{1}\right)\left(2\sigma_{XY}+V_{2}\right)}{\left(\mu_{X}^{2}+\mu_{Y}^{2}+V_{1}\right)\left(\sigma_{X}^{2}+\sigma_{Y}^{2}+V_{2}\right)}$$

where $\mu_{X}$ and $\mu_{Y}$ are local means; $\sigma_{X}^{2}$ and $\sigma_{Y}^{2}$ are local variances; $\sigma_{XY}$ is the covariance; and $V_{1}={\left(l_{1}{\mathrm{MAX}}_{I}\right)}^{2}$, $V_{2}={\left(l_{2}{\mathrm{MAX}}_{I}\right)}^{2}$ with *l*_1_ = 0.01, *l*_2_ = 0.03.

③ *FID*: Generated images with a lower *FID* indicate the generated distribution is closer to the reference. The *FID* is calculated using Equation (36).

(36) $$FID=\|\eta_{X}-\eta_{Y}{\|}^{2}+Tr(\sum_{X}+\sum_{Y}-2{\left(\sum_{X}\sum_{Y}\right)}^{1/2})$$

where $\left(\eta_{X},\Sigma_{X}\right)$ and $\left(\eta_{Y},\Sigma_{Y}\right)$ are the mean and covariance of deep features for the referenced and generated images, respectively.

The *PSNR*, *SSIM,* and *FID* of different methods are presented in Table 2.

It is evidence that the proposed FogGAN achieves higher *PSNR* and *SSIM* values while exhibiting lower *FID* compared with DCGAN, CycleGAN, and Pix2Pix. Since the generator in FogGAN is enhanced by injecting physical image cues, the generated images exhibit greater similarity to the original images in terms of structural features, with high values in *PSNR* and *SSIM*. Meanwhile, FogGAN improves the pixel-level fidelity and creates a more realistic road background with lower *FID*.

### 4.3. Agglomerate Fog Detection Results Based on the Fusion of SURF and Optical Flow

#### 4.3.1. SURF Extraction Results and Analysis

**Figure 8:**
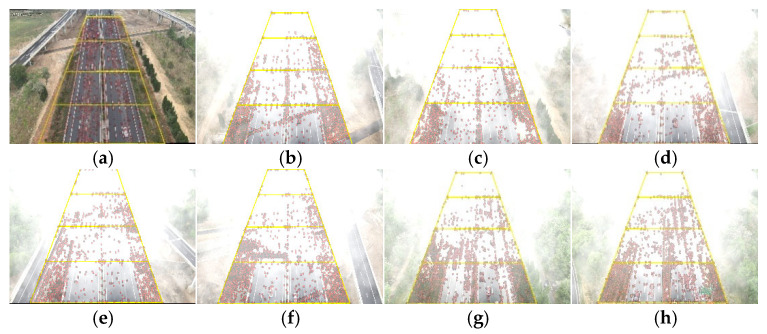
SURF extraction results: (**a**) Reference (clear) image. (**b**–**h**) Detection results under agglomerate fog, with yellow trapezoids marking road ROIs, and R1–R4 (near → far) and red dots indicating SURF key points. All panels use identical detector parameters and are aligned to the reference in (**a**).

In this paper, four ROIs are configured for agglomerate fog detection. The SURF extraction results for partial samples are shown in Figure 8.

**Figure 9:**
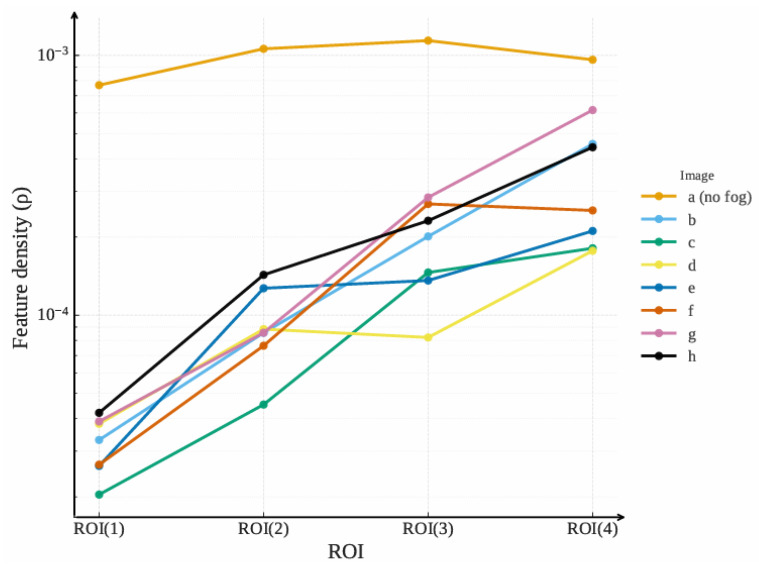
SURF density variation of different samples and ROIs.

The number of SURF $N_{f}$ and density ρ for different images and ROIs are presented in Table 3 and Figure 9.

Referring to Figure 8, Figure 9, and Table 3, the distribution of SURF points follows a pattern where the density is lower in long-distance ROIs and higher in short-distance ROIs. The reason is that agglomerate fog concentration is higher in long-distance regions, allowing the image to capture limited features that mainly originate from the agglomerate fog. In contrast, visibility is relatively better in short-distance regions, enabling ground targets to generate more feature points. Therefore, SURF features can effectively describe the state of agglomerate fog in an actual scenario.

#### 4.3.2. Optical Flow Extraction Results and Analysis

**Figure 10:**
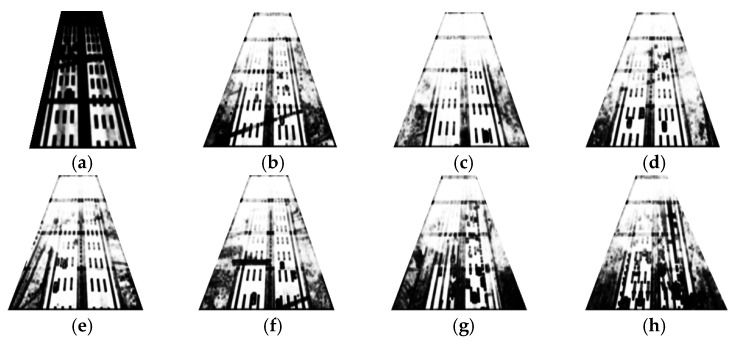
Optical flow extraction results: (**a**) Reference (clear) image. (**b**–**h**) Detection results under agglomerate fog. The visualization encodes magnitude only: darker = larger motion, brighter = smaller motion. All panels share the same contrast range for comparability and are aligned to the reference in (**a**).

Taking any two consecutive frames as examples, the optical flow is detected and is shown in Figure 10. In the figure, the optical flow magnitude is represented by the brightness.

The optical flow magnitude $M_{v}$ for different images and ROIs is presented in Table 4.

**Figure 11:**
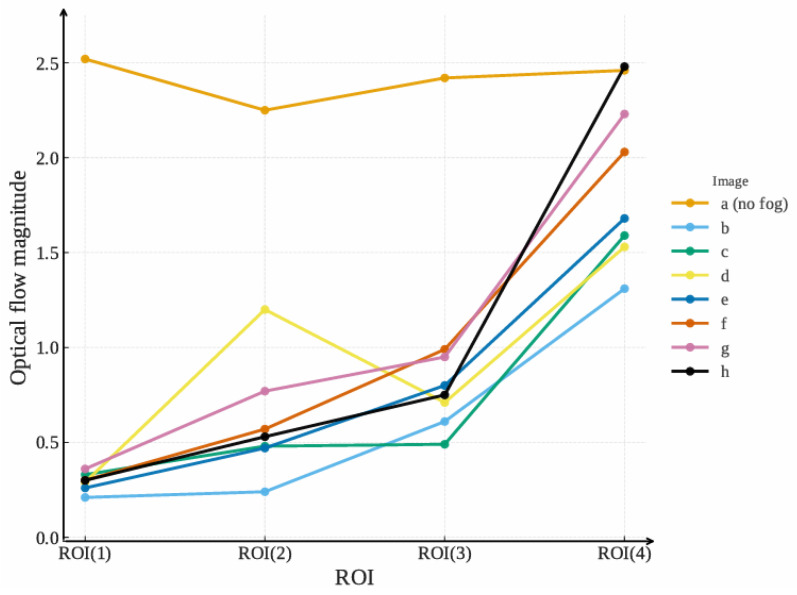
Optical flow magnitude variation of different samples and ROIs.

Referring to Figure 10, Figure 11, and Table 4, the optical flow magnitude exhibits a distribution pattern where values are smaller in long-distance ROIs and larger in short-distance ROIs. The reason is that higher agglomerate fog concentration in long-distance ROIs results in minimal pixel variation between consecutive frames captured by the moving UAV. In contrast, better visibility at close range leads to significant pixel changes due to dynamic UAV perspective shifts.

#### 4.3.3. Fusion Method Experimental Results and Analysis

Based on the aforementioned analysis, the SURF and optical flow features can be independently applied to the judgement of the agglomerate fog state by setting certain thresholds. Referring to Equations (28)–(30), the agglomerate fog state is detected for different images, including 300 training samples and 100 test samples. The precision, recall, and F1-score of the SURF-based method, optical flow-based method, and the fusion method are presented in Table 5.

It is evidence that both the SURF-based method and the optical flow-based method can achieve high precision for the agglomerate fog state judgement. However, the fusion of the two features presents better performance, where the precision increases by 7.3% compared to the SURF-based method and by 11.4% compared to the optical flow-based method; the recall improved by 13.3% and 9.0%, respectively; and the F1-score improved by 5.1% and 7.9%, respectively.

To further evaluate the performance of the proposed method, the XGBoost-based method [13] and the survey-informed fusion method [12] are selected for the comparison of agglomerate fog detection. All methods are tested under identical experimental settings using the same mixed dataset generated by FogGAN. The precision, recall, and F1-score are presented in Table 6.

The experimental results show that the proposed SURF and optical flow fusion method presents higher precision, recall, and F1-score compared with the XGBoost method and the survey-informed fusion method. Different from the XGBoost-based method, which relies on a dynamic light source, and the survey-informed fusion method, which requires high texture agglomerate fog images, the implement of the fusion of SURF and optical flow is able to detect both the static and dynamic physical characteristics of agglomerate fog in localized ROIs, and thus it yields better performance under rapidly changing viewpoints of UAVs. Since optical flow detection requires the comparison of two consecutive images, the CPU time is longer than that of the other two methods. Nevertheless, the millisecond-level additional CPU time will not substantially impact the practical engineering application of agglomerate fog detection; for example, the dissemination of fog status.

## 5. Conclusions and Future Work

This paper proposes an improved generative adversarial network (FogGAN) to obtain a high-quality and realistic agglomerate fog dataset, in which the generator is enhanced by injecting physical image cues into its feature modulation layers. Using the data sample generated by FogGAN, a fusion method of SURF and optical flow for detecting agglomerate fog in UAV-captured road scenarios is further presented. The method describes static features using SURF and dynamic features using optical flow magnitude, achieving better performance for agglomerate fog detection under the flight viewpoint of UAVs.

Future work includes the following: (1) Experiments will be further carried out under different UAV flight scenarios, such as different flight altitudes and camera resolutions, to evaluate the performance of the proposed method. (2) The integration of deep convolutional features of images with SURF and optical flow should be further explored to improve the accuracy of agglomerate fog detection.

## Figures and Tables

**Table 1:** Parameters of the experiment.

Category	Parameter	Value
Environmental conditions	wind scale	1–4 F
	temperature	10–25 °C
	visibility	4–10 km
UAV configurations	pitching angle	60°
	resolution of the camera	3840 × 2160 Px
	frame rate	30 FPS
	locate mode	RTK ± 0.1 m
Flight configurations	height	100–150 m
	speed	15 m/s

**Table 2:** *PSNR*, *SSIM,* and *FID* of different methods.

Method	*PSNR* (dB)	*SSIM*	*FID*
DCGAN	20.5	0.65	35.2
CycleGAN Pix2Pix FogGAN	22.3 23.1 24.1	0.70 0.72 0.77	30.1 28.4 25.3

**Table 3:** SURF distributions for different images and ROIs.

Image	ROI(1)		ROI(2)		ROI(3)		ROI(4)	
	$N_{f}$	ρ	$N_{f}$	ρ	$N_{f}$	ρ	$N_{f}$	ρ
a (no fog)	2711	7.68 × 10^−4^	6012	1.06 × 10^−3^	8910	1.14 × 10^−3^	9598	9.62 × 10^−4^
b	117	3.31 × 10^−5^	452	8.56 × 10^−5^	1411	2.01 × 10^−4^	3996	4.56 × 10^−4^
c	72	2.04 × 10^−5^	239	4.53 × 10^−5^	1024	1.46 × 10^−4^	1585	1.81 × 10^−4^
d	135	3.82 × 10^−5^	467	8.84 × 10^−5^	577	8.21 × 10^−5^	1552	1.77 × 10^−4^
e	93	2.63 × 10^−5^	670	1.27 × 10^−4^	957	1.36 × 10^−4^	1847	2.11 × 10^−4^
f	94	2.66 × 10^−5^	403	7.63 × 10^−5^	1884	2.68 × 10^−4^	2218	2.53 × 10^−4^
g	138	3.90 × 10^−5^	452	8.56 × 10^−5^	2000	2.84 × 10^−4^	5406	6.16 × 10^−4^
h	149	4.21 × 10^−5^	756	1.43 × 10^−4^	1622	2.31 × 10^−4^	3881	4.43 × 10^−4^

**Table 4:** Optical flow magnitude distribution for different images and ROIs.

Image	ROI(1)	ROI(2)	ROI(3)	ROI(4)
a (no fog)	2.52	2.25	2.42	2.46
b	0.21	0.24	0.61	1.31
c	0.33	0.48	0.49	1.59
d	0.29	1.20	0.71	1.53
e	0.26	0.47	0.80	1.68
f	0.30	0.57	0.99	2.03
g	0.36	0.77	0.95	2.23
h	0.30	0.53	0.75	2.48

**Table 5:** Agglomerate fog detection performance of the proposed method.

Method	Precision	Recall	F1-Score
SURF-based method	0.82	0.75	0.78
Optical flow-based method Fusion of SURF and optical flow method	0.79 0.88	0.78 0.85	0.76 0.82

**Table 6:** Agglomerate fog detection results of different methods.

Method	Precision	Recall	F1-Score	Processing Time (ms/Image)
XGBoost-based method	0.78	0.74	0.76	140
Survey informed fusion method Fusion of SURF and optical flow method	0.80 0.88	0.76 0.85	0.78 0.82	135 180

## Data Availability

The original contributions presented in this study are included in the article. Further inquiries can be directed to the corresponding author(s).
